# Plasminogen activator inhibitor-1 (PAI-1) expression in endometriosis

**DOI:** 10.1371/journal.pone.0219064

**Published:** 2019-07-17

**Authors:** Fahad T. Alotaibi, Bo Peng, Christian Klausen, Anna F. Lee, Amr O. Abdelkareem, Natasha L. Orr, Heather Noga, Mohamed A. Bedaiwy, Paul J. Yong

**Affiliations:** Department of Obstetrics & Gynaecology, BC Children’s Hospital Research Institute, The University of British Columbia, Vancouver, Canada; Universite du Quebec a Trois-Rivieres, CANADA

## Abstract

**Purpose:**

Deep infiltrating endometriosis (DIE) is defined as an endometriotic lesion penetrating to a depth of >5 mm and is associated with pelvic pain, but the underlying mechanisms are unclear. Our objective is to investigate whether plasminogen activator inhibitor-1 expression (PAI-1) in endometriotic tissues is increased in women with DIE.

**Methods:**

In this blinded *in vitro* study, immunohistochemistry and Histoscore were used to examine the expression of PAI-1 in glandular epithelium (GECs) and stroma (SCs) in a total of 62 women: deep infiltrating uterosacral/rectovaginal endometriosis (DIE; n = 13), ovarian endometrioma (OMA; n = 14), superficial peritoneal uterosacral/cul-de-sac endometriosis (SUP; n = 23), uterine (eutopic) endometrium from women with endometriosis (UE; n = 6), and non-endometriosis eutopic endometrium (UC; n = 6). The following patient characteristics were also collected: age, American Fertility Society stage, hormonal suppression, phase of menstrual cycle, dysmenorrhea score and deep dyspareunia score.

**Results:**

PAI-1 expression in GECs and SCs of the DIE group was significantly higher than that of SUP group (p = 0.01, p = 0.01, respectively) and UE group (p = 0.03, p = 0.04, respectively). Interestingly, increased PAI-1 expression in GECs and SCs was also significantly correlated with increased dysmenorrhea (r = 0.38, p = 0.01; r = 0.34, p = 0.02, respectively).

**Conclusions:**

We found higher expression of PAI-1 in DIE, and an association between PAI-1 and worse dysmenorrhea.

## Introduction

Endometriosis is a common, estrogen-dependent, chronic gynecological disorder associated with pelvic pain and infertility. It is characterized by the presence of uterine endometrial tissue (glandular epithelium and stroma) outside of its normal location—mainly on the pelvic peritoneum, but also on the ovaries and other pelvic organs, and more rarely in the pericardium, pleura, and even the brain [1, 2, 3]_._ Endometriosis-associated pelvic pain is multifactorial. In some cases, pain is related to deep infiltrating endometriosis (DIE) invading visceral organs that are associated with adhesions and/or large bulk of disease (e.g. large ovarian endometrioma cysts). DIE lesions have a specific anatomical distribution, as they are often found in the cul-de-sac and on the uterosacral ligaments.

The plasminogen activator (PA) system has been implicated in tumor invasion, compromising anatomical barriers and regulating the migration of tumor cells into adjacent normal tissues. Plasminogen activator inhibitor-1 (PAI-1) is a serine protease inhibitor (serpin) encoded by the human SERPINE1 gene. In addition to PAI-1 being associated with thrombosis/fibrosis (by inhibiting fibrinolysis), this inhibitor is involved in cell invasion and migration [4]. Local invasion and tumor vascularization of transplanted malignant keratinocytes was prevented in mice deficient of PAI-1, and was restored upon adenoviral vector-mediated expression of human PAI-1 [4]. Liu et al. [5] investigated the importance of the urokinase-type plasminogen activator (u-PA) system and PAI-1 in human lung cancer cell invasion. Inhibitory antibodies against u-PA and PAI-1 were found to suppress the invasion of H292 lung cancer cells. Immunohistochemical studies have demonstrated elevated PAI-1 levels in several cancers including breast cancer [6], endometrial cancer [7] and lung cancer [8]. Interestingly, the expression of PAI-1 was found to be significantly higher in women with stage III endometrial cancer compared to women with stage I or II [9].

Given that PAI-1 has been implicated in cell invasion in several contexts, the objective of this study was to determine whether PAI-1 expression is associated with DIE compared to other anatomic subtypes of endometriosis. We also sought to determine whether PAI-1 expression was associated with different pelvic pain symptoms, such as dysmenorrhea (menstrual cramps) and deep dyspareunia (pain with deep vaginal penetration). We hypothesized that increased PAI-1 expression would be associated with deep infiltrating endometriosis and increased pain.

## Materials and methods

### Setting

This study was carried out at an academic hospital-based program, the Centre for Pelvic Pain and Endometriosis, which is the tertiary referral center for British Columbia. All endometriosis cases involved women who underwent surgery to diagnose and excise endometriosis. Tissues were obtained from the pathology archives of Vancouver General Hospital and BC Women’s Hospital (2010–2017), either by prospective consent at surgery or by retrospective access with a waiver of consent (University of British Columbia Ethics approvals: H11-02882, H11-00536, H13-02563, and H14-03040).

The cohort consisted of 62 reproductive age women meeting one the following criteria: (a) deep infiltrating uterosacral/rectovaginal endometriosis (DIE, n = 13); (b) ovarian endometriomas (OMA, n = 14); (c) superficial peritoneal uterosacral/cul-de-sac endometriosis (SUP, n = 23); (d) uterine (eutopic) endometrium from women with a history of endometriosis (different from the women in groups a-c) (UE, n = 6); and (e) non-endometriosis eutopic endometrium from women undergoing endometrial sampling or hysterectomy for other clinical indications (UC, n = 6).

Some of these cases overlap with our previous studies: 23 SUP cases were previously used for work on nerve density and nerve growth factor in endometriosis;[10, 11] and 7 DIE cases, 7 OMA cases, and 6 UE cases have been previously used for studies on HOXB4 in endometriosis [12].

We also reviewed medical records and patient questionnaires (e.g. from pelvicpain.org and later modified as described in Yosef et al. [13]) to collect the following characteristics for each participant: age, American Fertility Society stage, hormonal suppression, phase of menstrual cycle, and 11-point numeric rating scales for dysmenorrhea score (0–10 severity) and deep dyspareunia score (0–10 severity) [14].

### Antibody validation

Specificity of the mouse monoclonal PAI-1 antibody (C-9, catalog no. sc-5297; Santa Cruz Biotechnology) was validated by small interfering RNA-mediated knockdown of PAI-1 in HepG2 liver carcinoma cells (S1 Fig). HepG2 cells (American Type Culture Collection) were cultured in Dulbecco's Modified Eagle's Medium (DMEM) supplemented with 10% Fetal bovine serum (FBS) and antibiotics (100 U/mL penicillin and 100 mg/mL streptomycin) at 37°C in a humidified atmosphere with 5% CO2. Cells were transfected for 48 hours with non-targeting control or human PAI-1 small interfering RNA (25 nmol/L; Qiagen) using Lipofectamine RNAiMAX Reagent (Life Technologies).

Knockdown was confirmed by reverse transcription quantitative real-time PCR performed with 96-well optical reaction plates utilizing an Applied Biosystems 7300 Real-Time PCR System (Thermo Fisher Scientific). Briefly, total RNA was extracted and 1 μg was used for first-strand cDNA synthesis with the Applied Biosystems High Capacity cDNA Reverse Transcription Kit (Thermo Fisher Scientific). Each 20 μL SYBR Green PCR reaction included 1×SYBR Green PCR Master Mix (Applied Biosystems), 25 ng cDNA, and 300 nmol/L of each primer. The primers used were: human PAI-1 (SERPINE1), 5’-CCTCAGGAAGCCCCTAGA-3’ (forward) and 5’-TGGAGAGGCTCTTGGTCT-3’ (reverse); and human glyceraldehyde-3-phosphate dehydrogenase (GAPDH), 5’-GAGTCAACGGATTTGGTCG-3’ (forward) and 5’-GACAAGCTTCCCGTTCTCAG-3’ (reverse). Relative quantification of mRNA levels was done by the comparative Cq method using GAPDH as the reference and the formula 2^−ΔΔCq^.

Immunocytochemistry was used to confirm specificity of the PAI-1 antibody by comparing PAI-1 staining in HepG2 cells with PAI-1 knockdown versus non-targeted control. Briefly, HepG2 cells were grown on coverslips, transfected with siRNA, fixed with ice-cold methanol, and permeabilized with 0.1% Tergitol-type NP-40 diluted in PBS. After incubation with Dako Dual Endogenous Enzyme Block for 10 minutes, coverslips were incubated overnight at 4°C with anti-PAI-1 antibody (1:25). Immunoreactivity was detected with the EnVision+ Dual Link system (Dako) and 3,3′-diaminobenzidine chromogen solution (Dako). The antibody was considered to be validated when PAI-1 expression was reduced in knocked down cells compared to the control cells (S1 Fig).

### Immunohistochemistry

Tissue biopsies from each patient in the cohort were fixed in 10% neutral buffered formalin, and processed into formalin-fixed, paraffin-embedded (FFPE) tissue blocks according to standard pathology laboratory procedure [15]. From each FFPE block, tissue sections of 4 microns in thickness were cut with a microtome and collected onto glass slides. One section was collected per patient. To prepare FFPE sections for immunohistochemistry, the endometrial sections were deparaffinized three times for 3 minutes in 100% xylene and rehydrated in a graded series of ethanol solutions (100%, 95%, 80% and 50%) for 3 minutes each. Wet heat-induced antigen retrieval was performed in a steamer for 30 minutes with a modified citrate buffer (pH 6.1; Dako). After incubation with Dako Dual Endogenous Enzyme Block for 10 min, slides were incubated overnight at 4°C with anti-PAI-1 antibody (1:25). Immunoreactivity was detected with the EnVision+ Dual Link system (Dako) and 3,3′-diaminobenzidine chromogen solution. Slides were counterstained with Harris hematoxylin (Sigma-Aldrich), dehydrated through graded ethanol to xylene, and coverslipped using a xylene-based mounting medium.

### Histoscore analysis

Immunohistochemical staining for PAI-1 in endometriotic epithelium and stroma was scored using the Histoscore method as previously described with some modifications[11] [12]. Two observers who were blinded to the group assignment independently scored one representative slide per case using a Leica light microscope. Areas of endometriosis epithelium and stroma were first scanned at low power (×10) and then analyzed at high power (×40) to evaluate the staining intensity and estimate the proportion of positive cells in up to 3 random fields per sample. Staining intensity was scored on a four-tiered scale (no staining = 0, low staining = 1, moderate staining = 2, and strong staining = 3) and the proportion of cells was determined for each staining intensity. Histoscores were calculated using the formula: Histoscore = 1 × proportion of cells with low staining + 2 × proportion of cells with moderate staining + 3 × proportion of cells with strong staining. A mean score from the two observers was calculated and intraclass correlation was 0.87, indicating high inter-observer reliability.

### Statistical analysis

Histoscore results are presented as mean ± standard deviation. The Kruskal-Wallis test was used to test for differences between all groups followed by the Mann-Whitney test for pairwise comparisons. Separate analyses were performed to compare PAI-1 Histoscores in the endometriotic glandular epithelial cells (GECs) and stroma cells (SCs). The Spearman rank correlation was used to investigate the correlation between PAI-1 Histoscore and clinical variables, such as severity of dysmenorrhea and deep dyspareunia. Statistical inference was guided by p-values < 0.05 and 95% confidence intervals. All statistical analyses were performed using SPSS 22.0 software (IBM Corporation, Armonk, New York).

## Results

For the cases in the five groups included in this study, the mean age was 34.2 ± 7.2 years (range 22–55). For the primary outcome (PAI-1 Histoscore), the mean score was 1.1 ± 0.8 in GECs and 0.9 ± 0.6 in SCs (range 0–3). For pain symptoms, the mean severity scores were 5.4 ± 3.7 (range 0–10) and 5.5 ± 4.1 (range 0–10) for dysmenorrhea and deep dyspareunia, respectively.

**Fig 1:**
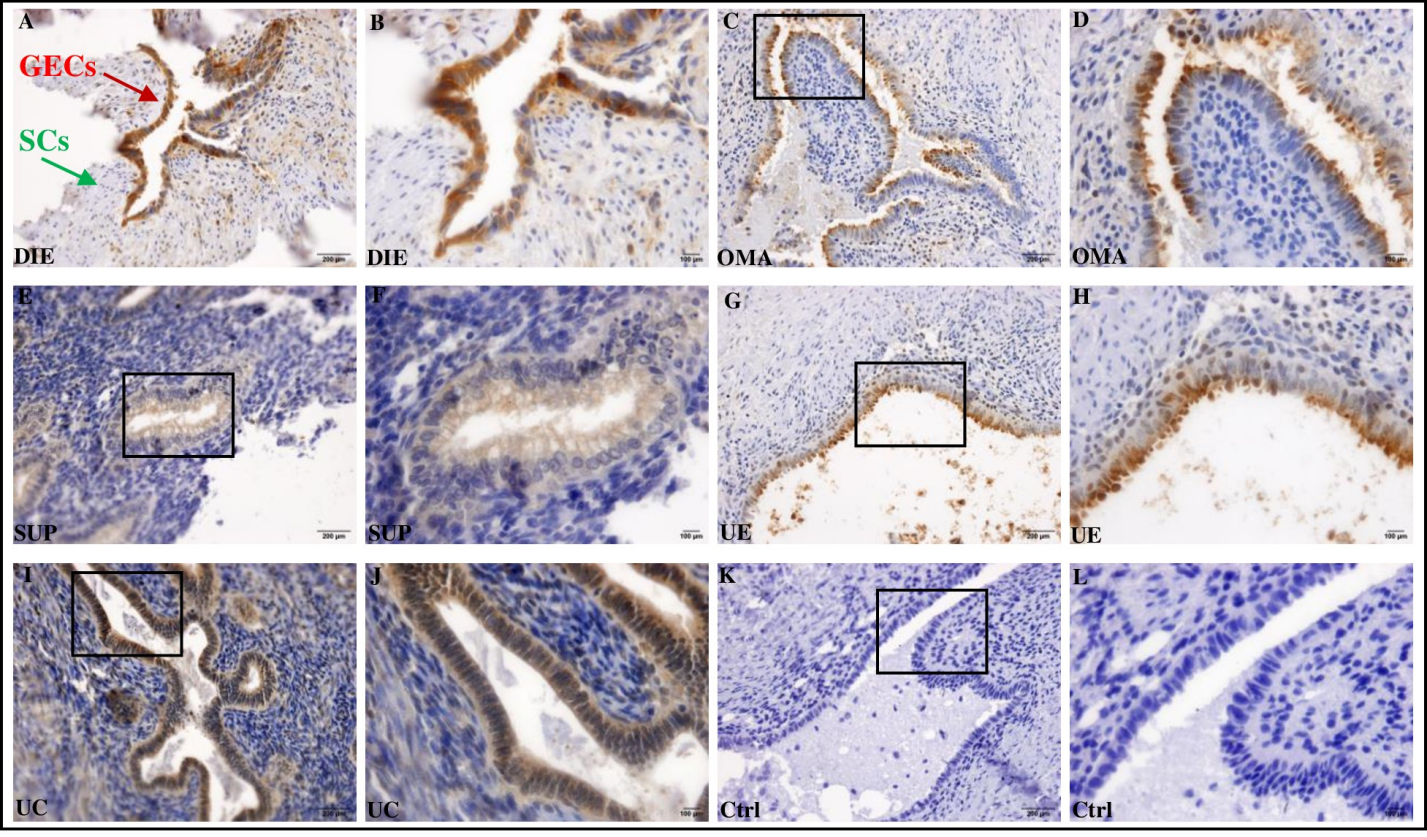
Immunohistochemical staining of PAI-1. PAI-1 staining in deep infiltrating uterosacral/rectovaginal endometriosis (DIE) (A and B), ovarian endometrioma (OMA) (C and D), superficial cul-de-sac/uterosacral endometriosis (SUP) (E and F), uterine (eutopic) endometrium (UE) (G and H), and non-endometriosis eutopic endometrium (UC) (I and J). No staining was observed in sections incubated without primary antibody (Ctrl) (K and L). GEC denotes glandular epithelial cells and SC denotes stromal cells. Original magnification 20×; Bar = 200 μm, 40×; Bar = 100 μm.

**Fig 2:**
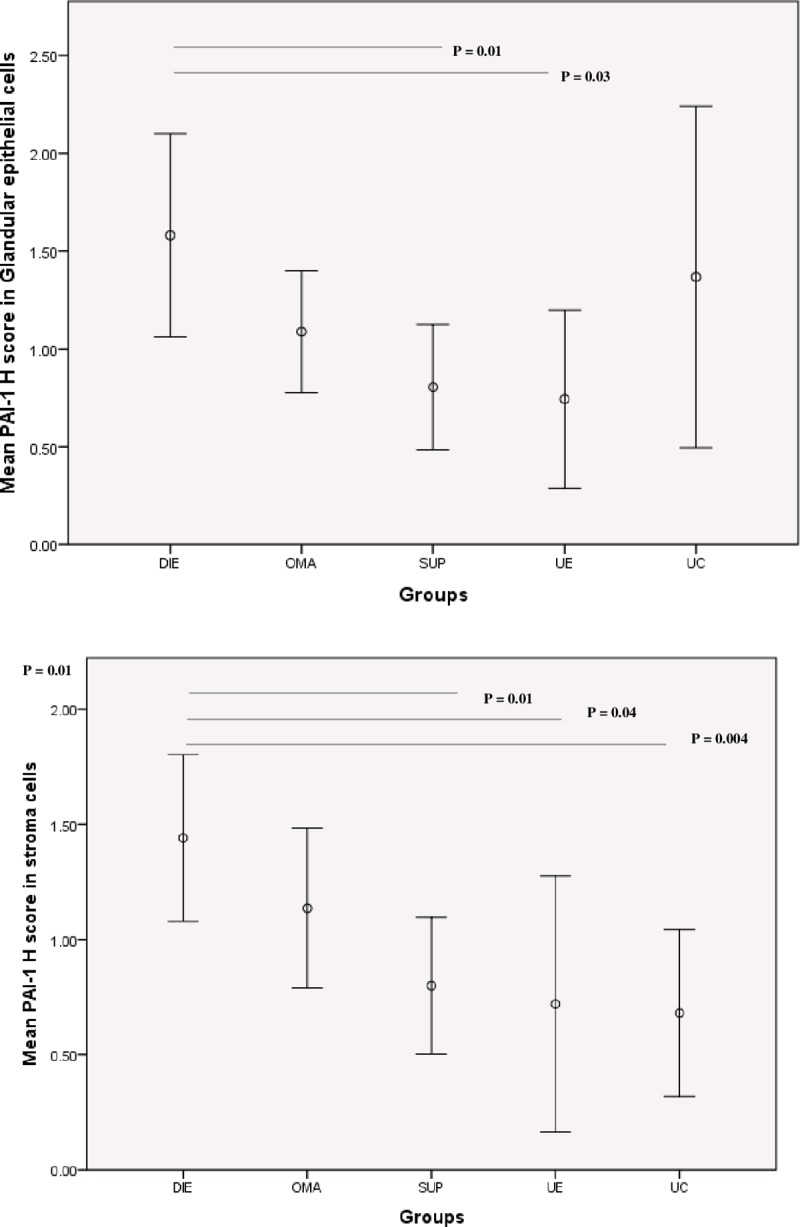
Analysis of PAI-1 Histoscores. PAI-1 Histoscores in glandular epithelial cells (upper panel) and stromal cells (lower panel) between deep infiltrating uterosacral/rectovaginal endometriosis (DIE, n = 13), ovarian endometrioma (OMA, n = 14), superficial peritoneal endometriosis (SUP, n = 23, uterine (eutopic) endometrium (UE, n = 6), and non-endometriosis eutopic endometrium (UC, n = 6).

Using the Histoscore (Figs 1 and 2), we found a difference between the five groups in PAI-1 expression in GECs and SCs (Kruskal-Wallis test, p = 0.04 for GECs and p = 0.03 for SCc). On pairwise comparison, PAI-1 expression in both in GECs and SCs in the DIE group was significantly higher than in the SUP group (p = 0.01, p = 0.01, respectively) and the UE group (p = 0.03, p = 0.04, respectively). In SCs only, PAI-1 expression was higher in DIE group when compared to UC group (p = 0.004).

**Fig 3:**
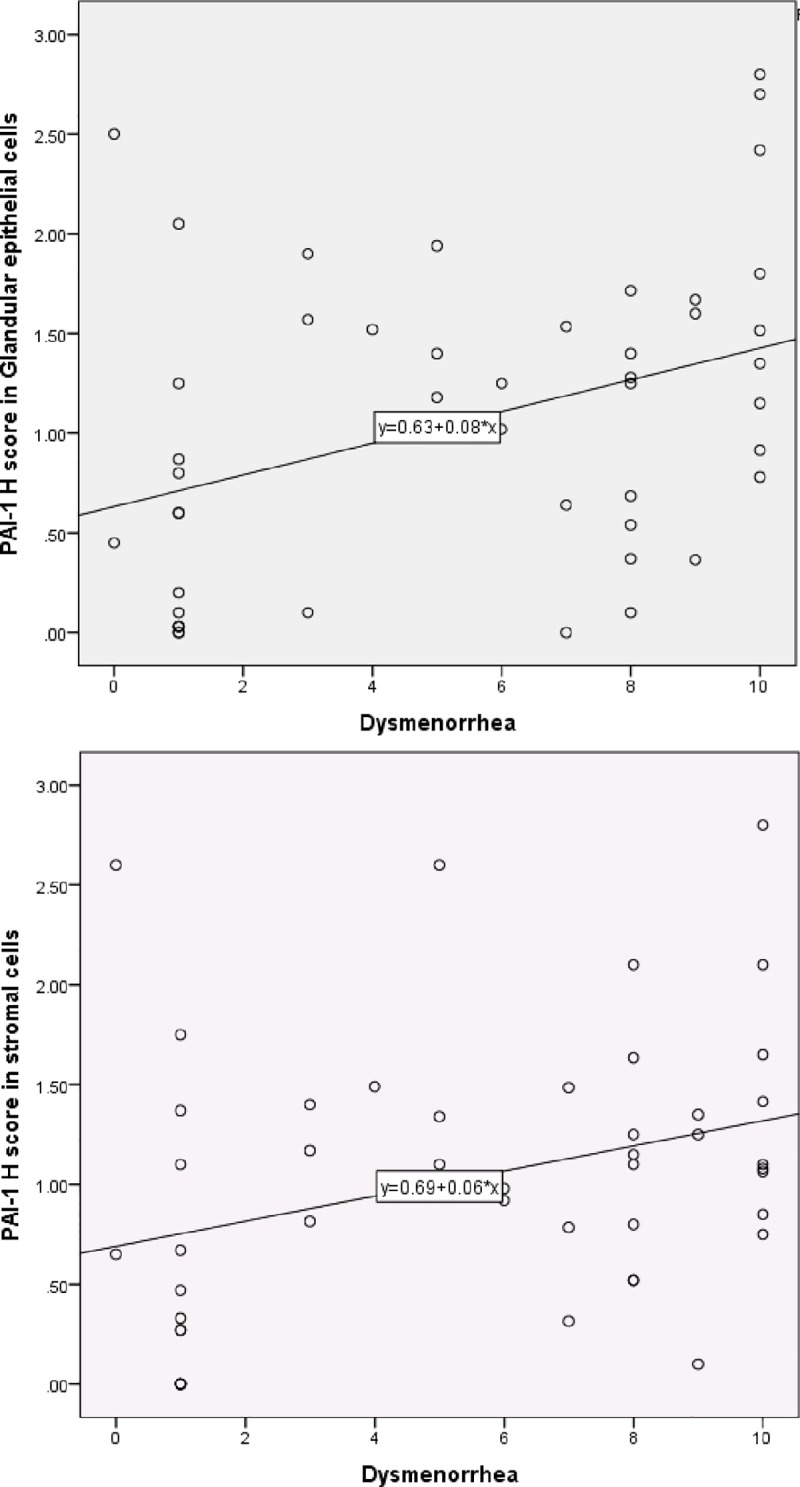
Scatterplot with a linear fit line of PAI-1 Histoscores and dysmenorrhea. The correlation between PAI-1 Histoscore and dysmenorrhea severity in glandular epithelial cells (r = 0.38, n = 46, p = 0.01) and stromal cells (r = 0.34, n = 46, p = 0.02).

We also tested for an association between PAI-1 expression and pain scores Table 1 (Fig 3). Interestingly, increased PAI-1 expression in GECs and SCs was significantly correlated with increased dysmenorrhea (r = 0.38, p = 0.01, r = 0.34, p = 0.02, respectively). A linear regression model was developed with severity of dysmenorrhea as the outcome variable, and PAI-1 expression in GECs, PAI-1 expression in SCs, and DIE (present or absent) as predictor variables. After backward elimination, only PAI-1 expression in GECs remained associated with dysmenorrhea (p = 0.001), suggesting that this association was independent of the presence or absence of DIE.

**Table 1:** Spearman’s correlation between PAI-1 Histoscore and intensities of dysmenorrhea and deep dyspareunia.

Spearman’s Correlation	Dysmenorrhea (0–10)			Deep dyspareunia (0–10)		
	Correlation Coefficient	Significance (2-tailed)	N	Correlation Coefficient	Significance (2-tailed)	N
PAI-1 Histoscore in endometriotic epithelium	0.38*	0.01	46	-0.30*	0.048	45
PAI-1 Histoscore in endometriotic stroma	0.34*	0.02	46	-0.26	0.08	45

* A significant value.

In contrast to dysmenorrhea, PAI-1 expression in GECs only was associated with decreased deep dyspareunia (r = -0.30, p = 0.048). There was no statistical correlation between PAI-1 expression, in both GECs and SCs, and other parameters such as hormonal suppression (p = 0.73, p = 0.31, respectively). (see S1 Table).

## Discussion

In this study, we found higher expression of PAI-1 in the DIE group compared to other endometriosis anatomic subtypes and eutopic endometrium. In particular, PAI-1 was significantly higher in DIE group compared to the SUP group and the UE group (GECs and SCs) and compared to the UC group (SCs only). Interestingly, increased PAI-1 expression was significantly correlated with increased dysmenorrhea (GECs and SCs), but decreased deep dyspareunia (GECs only).

The role of PAI-1 in the development of different diseases, including deep vein thrombosis [16], myocardial infarction[17], fibrotic disorders [18], metabolic disorders [19], and cancer[20], is well recognized. The ability of PAI-1 to impair peritoneal fibrinolysis could facilitate adhesion formation in endometriosis [21]. To the best of our knowledge, the association between PAI-1 and deep infiltrating endometriosis has not been studied yet. Our data showing increased PAI-1 expression in an invasive subtype of endometriosis is consistent with studies showing elevated PAI-1 in advanced (stage III) compared to low stage (I and II) endometrial cancer [9]. These findings support the involvement of PAI-1 in invasive processes in different diseases. Indeed, our finding of elevated PAI-1 expression in DIE compared to superficial endometriosis or uterine (eutopic) endometrium is not unexpected because invasion and fibrosis are characteristic of DIE. Equally, the lack of significant difference between the DIE group and the OMA group could reflect similar invasive or fibrotic properties of these cysts that invade the ovary and are associated with adhesions/fibrosis.

Our finding that increased PAI-1 expression is associated with dysmenorrhea could be explained by the role of PAI-1 in inflammation. Neuroinflammation is a significant factor in endometriosis-associated pain. PAI-1 is a pro-inflammatory factor with pro-thrombotic effects[21]. In adipose tissue, PAI-1 has been reported to be stimulated by TNF-α, an important pro-inflammatory cytokine[22] [23]. In contrast, we also found that PAI-1 expression in GECs was negatively correlated with severity of deep dyspareunia. This finding is consistent with published data showing a negative correlation between PAI-1 levels and sexual dysfunction[24]. Specifically, Veronelli et al. evaluated PAI-1, female sexual function, endocrine and metabolic parameters in diabetic, obese and hypothyroid women compared to healthy women[24]. They reported that PAI-1 was inversely correlated with female sexual function score and negatively predicted reduced female sexual function score (i.e. lower PAI-1 was associated with a higher score and worse sexual function). Future studies are needed to clarify the relationships between PAI-1, pelvic pain, and sexual function. As well, it would be interesting to perform similar analyses with other components of the plasminogen activator system, such as u-PA or tissue-type plasminogen activator (t-PA).

The strengths of our study are the use of a validated antibody, and assessment of anatomical and pain outcomes. In addition, in order to avoid bias, immunohistochemistry experiments were carried out blinded to clinical group. One limitation of the study is that only one representative slide per case was examined. Also, we utilized only PAI-1 antibody, and so we do not have data on t-PA or u-PA expression. Further, in this retrospective study, we did not systematically collect data on other pain contributors (e.g. adenomyosis or pelvic congestion syndrome), and thus we are not certain whether such conditions are influencing the dysmenorrhea score in our sample.

The association between PAI-1 and DIE points to future work to examine whether PAI-1 expression drives proliferation or invasion in endometriosis cells. If so, PAI-1 could serve as a potential drug target for the management of endometriosis; recently, it was found that treatment with the small molecule PAI-1 inhibitor TM5275 effectively blocked the proliferation of ovarian cancer cells with high expression of PAI-1[25]. It was suggested that PAI-1 inhibition promoted cell cycle arrest and apoptosis in ovarian cancer, and that PAI-1 inhibitors may represent a novel class of antitumor agents. Small molecule inhibitors of PAI-1 (TM5275 and TM5441) have also been shown to reduce the viability of several other human cancer cell lines[26]. Future studies investigating the effects of forced-expression or knockdown of PAI-1 on the invasiveness or proliferation of primary endometriotic cells will be of great interest. Additional studies could explore the mechanisms by which PAI-1 may promote cell invasion in endometriosis, such as the up-regulation of integrin α5 and vascular endothelial growth factor (VEGF) expression [27].

## Supporting information

S1 Fig Knockdown and immunostaining of PAI-1 in HepG2 cells. Human SERPINE1 siRNA (siPAI-1) and Control siRNA were used for siRNA experiment. Cells were fixed and immunostained with the monoclonal PAI-1 antibody at dilution 1:25, which showed staining in the control siRNA HepG2 cells but much less staining in the siPAI-1 knockdown HepG2 cells.
(TIF)

S1 Table PAI-1 Histoscores analysis. (DOCX)
