# Supplementary figures and images for: Plasminogen activator inhibitor-1 (PAI-1) expression in endometriosis
Source: PLoS One. 2019 Jul 17;14(7):e0219064. doi: 10.1371/journal.pone.0219064 (PMC6637014; doi:10.1371/journal.pone.0219064)

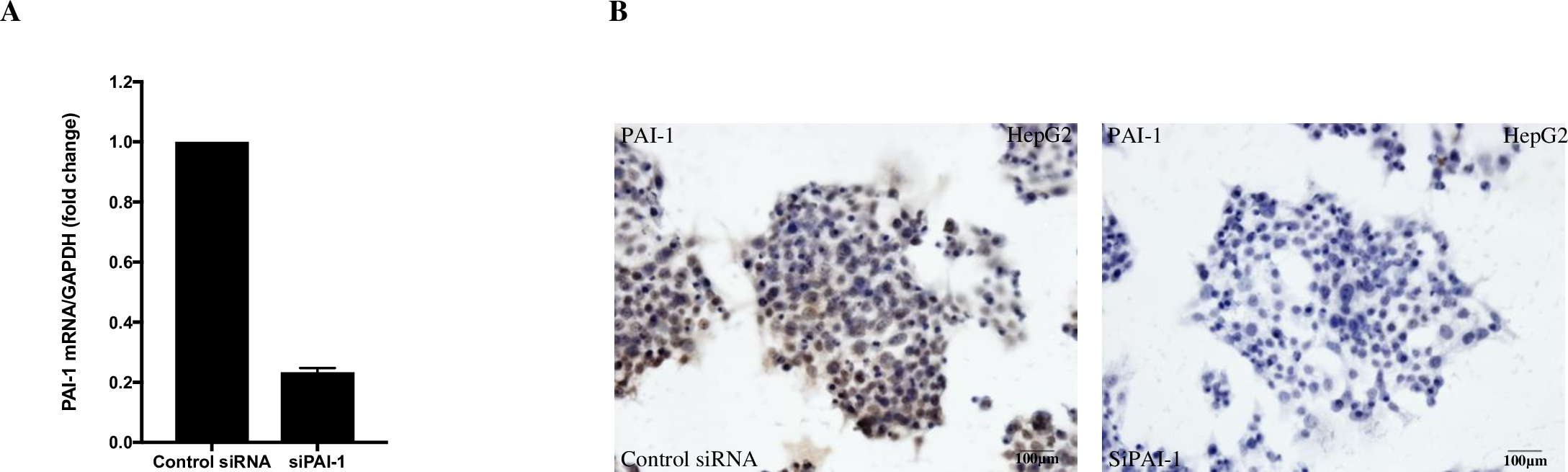

Supplement: S1 Fig — Human SERPINE1 siRNA (siPAI-1) and Control siRNA were used for siRNA experiment. Cells were fixed and immunostained with the monoclonal PAI-1 antibody at dilution 1:25, which showed staining in the control siRNA HepG2 cells but much less staining in the siPAI-1 knockdown HepG2 cells. (TIF) [file pone.0219064.s001.tif]
