# Supplementary material for: Plasminogen activator inhibitor-1 (PAI-1) expression in endometriosis
Source: PLoS One. 2019 Jul 17;14(7):e0219064. doi: 10.1371/journal.pone.0219064 (PMC6637014; doi:10.1371/journal.pone.0219064)
Supplement: S1 Table — (DOCX) [file pone.0219064.s002.docx]

**S1 Table**

Other pairwise comparisons of PAI-1 expression in GECs and SCs between endometriosis groups were not significantly different:

| Groups | PAI-1 Histoscore in endometriotic epithelium |
| --- | --- |
|  | P value |
| DIE vs OMA | 0.06 |
| DIE vs UC | 0.58 |
| OMA vs SUP | 0.19 |
| OMA vs UE | 0.20 |
| OMA vs UC | 0.28 |
| SUP vs UE | 0.87 |
| SUP vs UC | 0.12 |
| UE vs UC | 0.15 |

| Groups | PAI-1 Histoscore in endometriotic stroma |
| --- | --- |
|  | P value |
| DIE vs OMA | 0.13 |
| OMA vs SUP | 0.19 |
| OMA vs UE | 0.39 |
| OMA vs UC | 0.21 |
| SUP vs UE | 0.91 |
| SUP vs UC | 0.83 |
| UE vs UC | 0.75 |

Furthermore, there was no statistical correlation between PAI-1 expression, in both GECs and SCs, and other clinical parameters including age (r = 0.06, p = 0.62, r = 0.09 p = 0.46, respectively), stages (Stage 1 vs 2: p = 0.66, p = 0.46. Stage 1 vs 3: p = 0.93, p = 0.85. Stage 1 vs 4: p = 0.70, p = 0.82. Stage 2 vs 3: p = 0.89, p = 0.42. Stage 2 vs 4: p = 0.93, p = 0.62. Stage 3 vs 4: p = 0.63, p = 0.72, respectively), hormonal suppression (p = 0.73 and p = 0.31), or menstrual cycle phases (proliferative vs secretory: p = 0.96, p = 0.56, respectively).
